# Temporal stability of functional brain modules associated with human intelligence

**DOI:** 10.1002/hbm.24807

**Published:** 2019-10-06

**Authors:** Kirsten Hilger, Makoto Fukushima, Olaf Sporns, Christian J. Fiebach

**Affiliations:** ^1^ Department of Psychology Goethe University Frankfurt Frankfurt am Main Germany; ^2^ IDeA Center for Individual Development and Adaptive Education Frankfurt am Main Germany; ^3^ Department of Psychological and Brain Sciences Indiana University Bloomington Indiana; ^4^ Brain Imaging Center Goethe University Frankfurt Frankfurt am Main Germany

**Keywords:** dynamic networks, graph theory, intelligence, modularity, resting‐state fMRI

## Abstract

Individual differences in general cognitive ability (i.e., intelligence) have been linked to individual variations in the modular organization of functional brain networks. However, these analyses have been limited to static (time‐averaged) connectivity, and have not yet addressed whether dynamic changes in the configuration of brain networks relate to general intelligence. Here, we used multiband functional MRI resting‐state data (*N* = 281) and estimated subject‐specific time‐varying functional connectivity networks. Modularity optimization was applied to determine individual time‐variant module partitions and to assess fluctuations in modularity across time. We show that higher intelligence, indexed by an established composite measure, the Wechsler Abbreviated Scale of Intelligence (WASI), is associated with higher temporal stability (lower temporal variability) of brain network modularity. Post‐hoc analyses reveal that subjects with higher intelligence scores engage in fewer periods of extremely high modularity — which are characterized by greater disconnection of task‐positive from task‐negative networks. Further, we show that brain regions of the dorsal attention network contribute most to the observed effect. In sum, our study suggests that investigating the temporal dynamics of functional brain network topology contributes to our understanding of the neural bases of general cognitive abilities.

## 1 INTRODUCTION

Intelligence describes our ability to reason, to understand complex ideas, to learn from experiences, and to adapt effectively to the environment (Neisser et al., 1996). Understanding the biological bases of human intelligence is an important scientific aim, and neuroscientific research has begun to contribute insights about how individual differences in brain function (Duncan, 2005; Sripada, Angstadt, & Rutherford, 2018), brain structure (Gregory et al., 2016; Haier, Jung, Yeo, Head, & Alkire, 2004), and intrinsic brain connectivity (Hilger, Ekman, Fiebach, & Basten, 2017a; Van den Heuvel, Stam, Kahn, & Hulshoff Pol, 2009) relate to general intelligence (for review see Basten, Hilger, & Fiebach, 2015; Jung & Haier, 2007).

Recent years have seen an increasing interest in understanding how human cognition emerges from the intrinsic organization of functional brain networks (Park & Friston, 2013), often studied using functional MRI (fMRI) in the absence of task demands (i.e., under resting‐state conditions; Biswal, Yetkin, Haughton, & Hyde, 1995). The topology of these networks determines how information is transferred between brain regions, and graph theory provides a set of tools to study these topological characteristics (Rubinov & Sporns, 2010). In the field of intelligence research, early graph‐theoretical work proposed that global properties of brain networks such as higher global network efficiency are associated with higher intelligence (van den Heuvel et al., 2009), a finding not replicated in more recent studies (Kruschwitz, Waller, Daedelow, Walter, & Veer, 2018; Pamplona, Santos Neto, Rosset, Rogers, & Salmon, 2015). In contrast, other studies have suggested that intelligence is related to efficiency in the interconnections of specific brain regions (Hilger et al., 2017a). Graph‐theoretical investigations revealed further that the human brain exhibits a hierarchically modular organization with clusters of nodes (modules, subnetworks) that are densely connected among each other but only sparsely coupled to nodes in other modules (Meunier, Lambiotte, & Bullmore, 2010; Sporns & Betzel, 2016). A modular organization balances segregated and integrated information processing, both of which are important for human cognition (Cohen & D'Esposito, 2016). Region‐specific modularity was recently also shown to covary significantly with individual differences in general intelligence (Hilger, Ekman, Fiebach, & Basten, 2017b).

The functional brain network correlates of intelligence were so far mostly studied as a static (i.e., time‐invariant) property of the human brain, that is, by averaging time courses of neural activation across the entire duration of a resting‐state fMRI scan (typically 5–10 min). This approach, however, ignores that intrinsic brain networks vary substantially across time (Cohen, 2018; Lurie et al., 2018; Zalesky, Fornito, Cocchi, Gollo, & Breakspear, 2014). Importantly, it has been shown that the dynamic interplay between states of high integration (low modularity) versus high segregation (high modularity) is linked to different levels of attention (Shine, Koyejo, & Poldrack, 2016) and cognitive performance (Shine et al., 2016). These first results suggest that the study of network dynamics has great potential for providing insights into human cognition from a mechanistic point of view — and thus also for advancing our understanding about the neural mechanisms underlying different levels of general cognitive ability.

Here, we apply graph‐theoretical modularity analyses to resting‐state BOLD fMRI data from a large sample of healthy adult humans (*N* = 281) to test the hypothesis that intelligence covaries significantly with the amount of dynamic reconfiguration within modularly organized, intrinsic brain networks. Going beyond previous work, we measured global modularity at different spatial scales, to gain insights into the brain's intrinsic network architecture beyond an arbitrarily chosen resolution level. The results of this analysis replicate and extend our previous finding that intelligence is not related to global modularity of static (i.e., time‐invariant) networks (Hilger et al., 2017b). Most importantly, we observed an association between intelligence and dynamic network reconfiguration, such that more intelligent persons show greater stability of network segregation over time.

## 2 METHODS

### 2.1 Participants

The data used in the current study were acquired by the Nathan S. Kline Institute for Psychiatric Research (Enhanced NKI Rockland sample, Release 1‐5; Nooner et al., 2012; http://fcon_1000.projects.nitrc.org/indi/enhanced/; NKI‐RS Enhanced Sample, RRID:SCR_010461). Procedures were approved by the NKI Institutional Review Board (#239708) and informed written consent was obtained from all participants. All analyses were based on a subsample of 281 healthy participants (98 males, mean age: 47.19 years, 246 right‐handed) for whom complete multiband neuroimaging and phenotypical data were available, including the Wechsler Abbreviated Scale of Intelligence (Wechsler, 1999; range: 69–141; mean FSIQ: 101.44), and whose imaging data sets survived the Connectome Computation System (CSS) quality check (see below).

### 2.2 Data acquisition and analysis

Fast sampling (TR = 645 ms) task‐free fMRI (eyes open) was acquired with a 32‐channel head coil on a 3T Siemens Tim Trio scanner. Acquisition parameters of the 9:46 min (≈900 time points) scans were: TE = 30 ms, flip angle = 60°, voxel size = 3 mm isotropic, FOV = 222 × 222 mm^2^, and 40 slices. A T1‐weighted structural scan (TR = 1,900 ms, TE = 2.52 ms, flip angle = 9°, voxel size = 1 mm isotropic, FOV = 250 × 250 mm^2^, 176 slices) was obtained from each participant for coregistration. Preprocessing was based on the CSS pipeline (Xu, Yang, Jiang, Xing, & Zuo, 2015; https://github.com/zuoxinian/CCS; RRID:SCR_017342) and involved discarding the first 16 volumes (10:32 s), removal and interpolation of outlier volumes (due to either hardware instability or head motion), slice timing and motion correction, global mean intensity normalization, coregistration between functional and structural images, nuisance regression using global, white matter, and CSF mean signals as well as 24 motion parameters (six motion parameter of the current and the preceding volume, plus each of these values squared; Friston, Williams, Howard, Frackowiak, & Turner, 1996), temporal band‐pass filtering (0.01–0.1 Hz), removal of linear and quadratic trends, and projection of the preprocessed time series onto a standard volume (MNI152). The low‐cut frequency of the temporal filtering (0.01 Hz) was specified as the reciprocal of the width of the time window (≈100 s, 156 time points). Nineteen participants from the initial sample of *N* = 300 were excluded by the CCS quality check due to low‐quality anatomical images, mean framewise displacement (*FD*) > 0.2 mm, maximum translation > 3 mm, maximum rotation > 3°, or minimum cost of boundary‐based registration > 0.6 (Greve & Fischl, 2010). Our approach to remove outliers is essentially equivalent to motion scrubbing (Power, Barnes, Snyder, Schlaggar, & Petersen, 2012) and strict censoring (Power et al., 2014; Siegel et al., 2017). However, instead of removing respective time points, we replaced outliers with an interpolated value (see, e.g., Siegel et al., 2017) to retain the same number of time points in all sliding windows (Betzel, Fukushima, He, Zuo, & Sporns, 2016; Fukushima et al., 2018). Detection of outliers and interpolation were performed using the function 3dDespike in AFNI (Allen et al., 2014; Cox, 2012; https://afni.nimh.nih.gov/pub/dist/doc/program_help/3dDespike.htm; RRID:SCR_005927).

### 2.3 Graph‐theoretical modularity analyses

**Figure 1:**
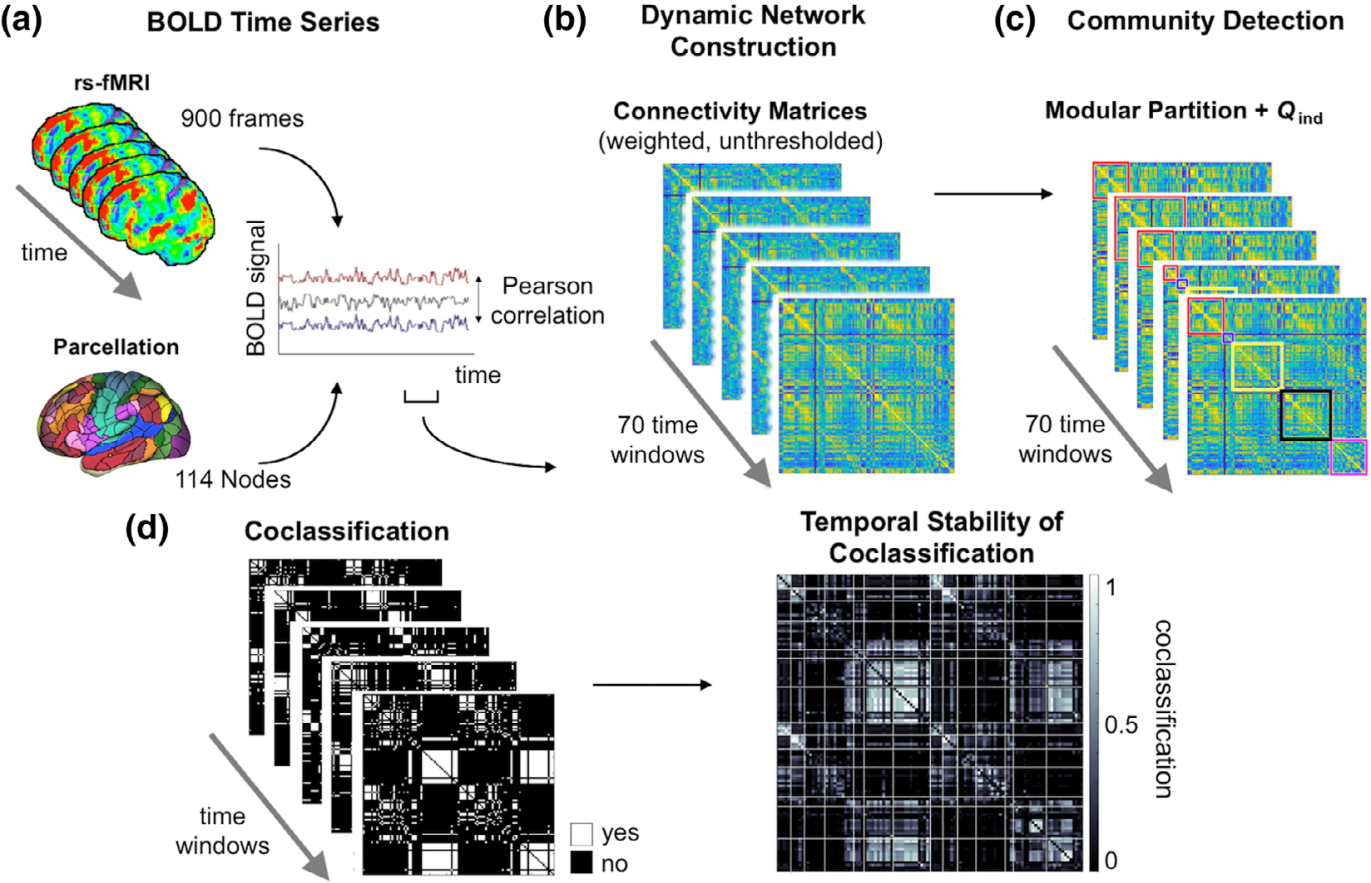
Schematic illustration of analysis workflow. (a) Multiband fMRI data was acquired during resting state. Node parcellation is based on 114 subdivisions of the 17‐network Yeo atlas (see Section 2). Edges were modeled on the basis of Pearson's correlation coefficients between node‐specific BOLD time series for 70 time windows of 156 time points. (b) Weighted unthresholded connectivity matrices were computed for each time window. (c) Community detection was performed on each connectivity matrix. (d) Left: window‐specific coclassification matrices indicating whether a node pair is assigned to the same module (white) at a given time point. Right: connection‐specific stability values were calculated as proportion of time windows in which two nodes are coclassified to the same module. High values (white) indicate that the nodes of a pair are assigned consistently to the same module (exhibiting stable consistent module affiliation). Low values (black) indicate that a node pair is only rarely coclassified to the same module; rather, nodes stay consistently segregated from each other which may be interpreted as “stable absence” of segregation. *Q*_*ind*_, global modularity of individual‐specific module partitions. fMRI, functional MRI [Color figure can be viewed at wileyonlinelibrary.com]

The cortical volume of the template brain was parcellated into 114 regions of the Yeo atlas (Figure 1a; Betzel et al., 2016; Yeo et al., 2011) that served as network nodes and allowed the assignment to 17 (used for the identification of individually optimal gamma values) or 7 (used for illustrating connection‐specific stability values as depicted in the coclassification matrix; cf. Figure 1d and 4a) functional networks (VIS, visual network; SMN, somatomotor network; DAN, dorsal attention network; VAN, ventral attention network; LIM, limbic network; CON, control network; DMN, default‐mode network; Yeo et al., 2011). Weighted edges were modeled on the basis of Fisher *z*‐transformed Pearson correlation coefficients between the nodes' BOLD time series (a) across the entire duration of the functional scan (analysis of static networks), and (b) within tapered (Fukushima et al., 2018) sliding time windows of length ≈100 s (156 time points; analysis of dynamic networks, see Figure 1b). Window length was determined so that windows capture the full cycle of the slowest frequency components (Betzel et al., 2016). Time windows were shifted across the BOLD time series by 10 time points, which resulted in a total of 70 partially overlapping windows. All analyses were also repeated using coarser window parcellations, without changes in our principal findings. Subject‐specific module partitions were determined by running the Louvain algorithm (Blondel, Guillaume, Lambiotte, & Lefebvre, 2008) 100 times for each of 60 spatial resolution levels (0.1 < *γ* ≤ 6; 0.1 steps) on (a) the correlation matrix of the static network and (b) the correlation matrices of each time window for the dynamic network analysis (Figure 1c). For each repetition of the Louvain algorithm, the single partition that maximizes global modularity *Q*_*ind*_ was selected, per gamma, per subject, and time window. All analyses focused on the resolution level (*γ* ) where subject‐specific static module partitions demonstrated highest agreement (mutual information) with the 17‐network partition of Yeo et al. (2011). Temporal variability of functional network organization was operationalized as the *SD* of *Q*_*ind*_ over time. Individual‐specific states of high and low modularity were defined as time windows where *Q*_*ind*_ exceeds or falls below the individual‐specific thresholds of mean *Q*_*ind*_ ± 50% of mean *Q*_*ind*_. This analysis was repeated using the modularity mean at the group‐level, that is, *Q* averaged across all time points and all participants, *Q*_*group*_. Connection‐specific stability values were computed as time‐averaged coclassification scores representing the proportion of time windows in which a given node pair is assigned (coclassified) into the same module (see Figure 1d and 4a,b, and Section 3 for further information). Network‐specific stability scores were determined by averaging the coclassification scores of all connections within and between the seven standard networks as specified by Yeo et al. (2011).

### 2.4 Individual difference analyses of network dynamics

Associations between graph measures and intelligence were calculated as partial correlations (Spearman, *rho*) controlling for effects of age, sex, handedness, and mean framewise displacement. Although not correlated with intelligence in the current sample (*rho* = −.08, *p* = .200), in‐scanner head motion is controlled for because it can produce spurious correlations between time series of neural activation and thus artificially introduce functional connections (Power et al., 2014) or associations with behavioral variables (Siegel et al., 2017). *p*‐values < .05 were interpreted as indicating statistical significance, except in cases of multiple comparisons, where *p*‐values were Bonferroni corrected (see below). To investigate the association between network‐specific stability values and intelligence (28 comparisons) *p*‐values of partial correlations were Bonferroni corrected, resulting in a corrected threshold of *p* < .0018. All analyses were conducted in Matlab (Version 2018a; MathWorks, Inc., Natick, MA; https://de.mathworks.com/products/matlab.html; RRID:SCR_001622).

## 3 RESULTS

### 3.1 Static network modularity and intelligence

We first focused on the analysis of static brain networks. To this end, we computed subject‐specific resting‐state functional connectivity across the duration of the entire fMRI scan (9:46 min), and extracted for each participant optimal modular partitions and global modularity scores (*Q*) by using modularity maximization (Blondel et al., 2008; Newman & Girvan, 2003). We varied the resolution parameter *γ* between 0.1 and 6.0, in steps of 0.1, to capture modules at different spatial scales. Selecting a single partition for each subject that best matched canonical resting‐state networks (Yeo et al., 2011), we replicated a previous result (Hilger et al., 2017b) indicating that individual differences in intelligence are not associated with variations in global modularity *Q* (Spearman's *rho* = −.03, *p* = .681). Extending previous results, we here also show that there is no association between intelligence and global modularity at any level of the resolution parameter *γ* (all *p >* .098; see Figure S1).

### 3.2 Network dynamics and intelligence

**Figure 2:**
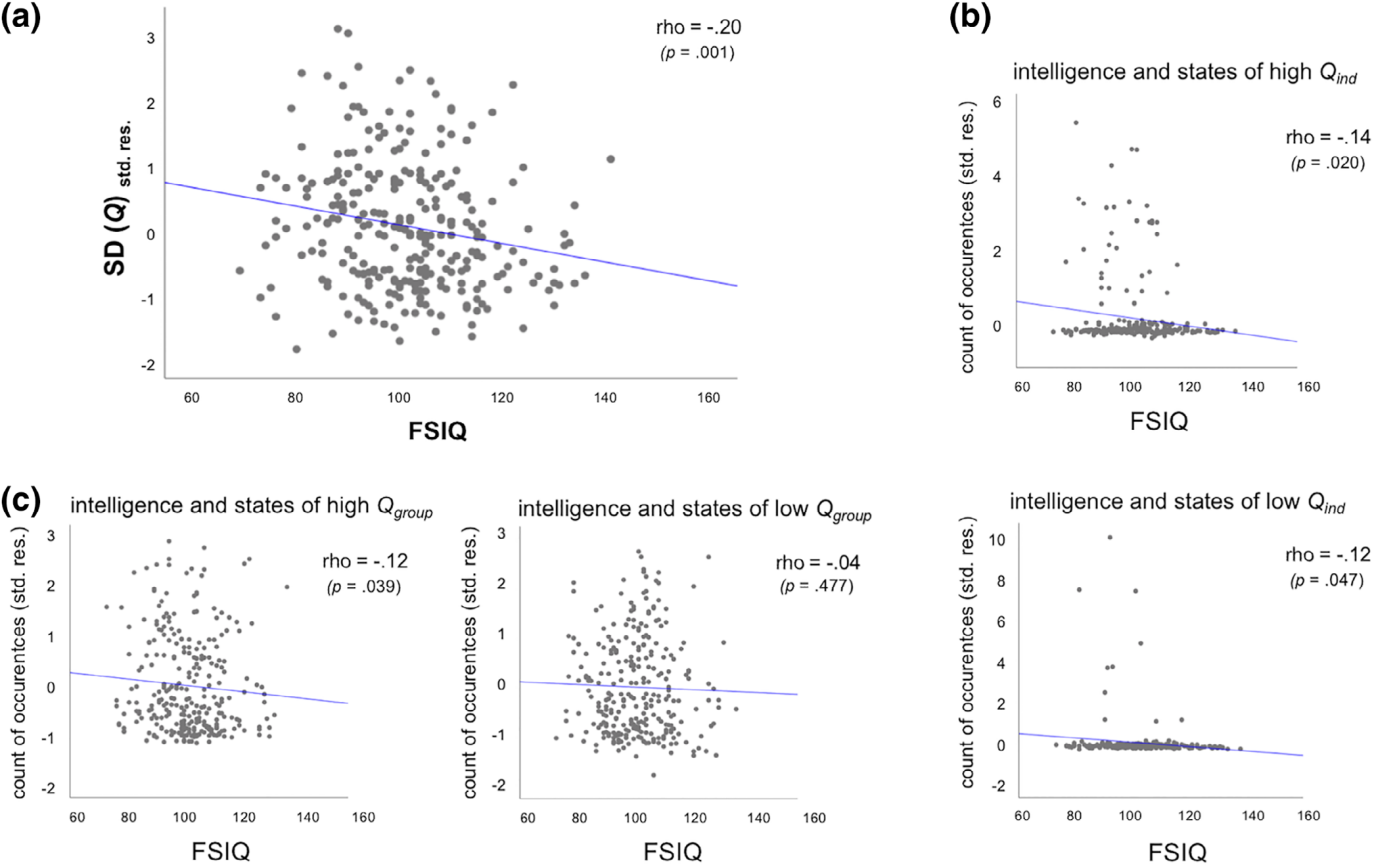
Scatterplots for the associations between intelligence and functional brain network characteristics controlling for effects for age, sex, handedness, and mean framewise displacement (*N* = 281). (a) Association between intelligence (and the inverse of) brain network stability as indexed by fluctuations over time in global modularity. Variability in global modularity was operationalized as the *SD* of *Q*_*ind*_ across time. (b) Association between intelligence and the number of subject‐specific high and low modularity states. States of high (upper row) and low (bottom row) modularity were operationalized relative to subject‐specific mean modularity (see Section 3 for more details). (c) Association between intelligence and the number of high (left) and low (right) modularity states defined in relation to the group‐averaged mean modularity. All illustrations represent partial correlations, with the *y*‐axis depicting the standardized residuals resulting from linear regression of age, sex, hand, and mean framewise displacement on the variable of interest, that is, *SD*(*Q*) or count of occurrences of modularity states. FSIQ, full scale intelligence quotient assessed with Wechsler Abbreviated Scale of Intelligence (WASI; Wechsler, 1999); *p*, *p‐*value of respective association indicating statistical significance if *p* < .05; *rho*, Spearman correlation coefficient; *SD*(*Q*), *SD* of global modularity from individual‐specific module partitions; std. res., standardized residuals [Color figure can be viewed at wileyonlinelibrary.com]

The primary aim of the current study was to examine the relationship between intelligence and the temporal dynamics of brain network reconfiguration, as indexed by fluctuations in global modularity. We observed that the *SD* of global modularity *Q*_*ind*_ over time (calculated for each participant at her or his optimal resolution level) varied considerably between individuals (range: .0083–.1011; *M* = .0361; *SD* = .0147) and, most importantly, that these fluctuations were significantly correlated with intelligence (*rho* = −.20, *p* = .001, *R* ^*2*^ = .04; see Figure 2a). This negative relationship was robust across a broad range of resolution levels (see Figure S1). The analysis was also repeated with individual's variability in the number of modules (*SD*_*num*_) added as an additional control variable. This does not change the results (correlation between IQ and variability in modularity over time with additional control variable; *r* = −.21, *p* = .0005). Thus, while subjects with higher intelligence scores did not exhibit different levels of segregation or integration in their time‐averaged functional networks (static network analyses; see previous section and Hilger et al., 2017a, 2017b), the level of segregation of their functional networks (as indexed by global modularity) varied significantly less over time.

**Figure 3:**
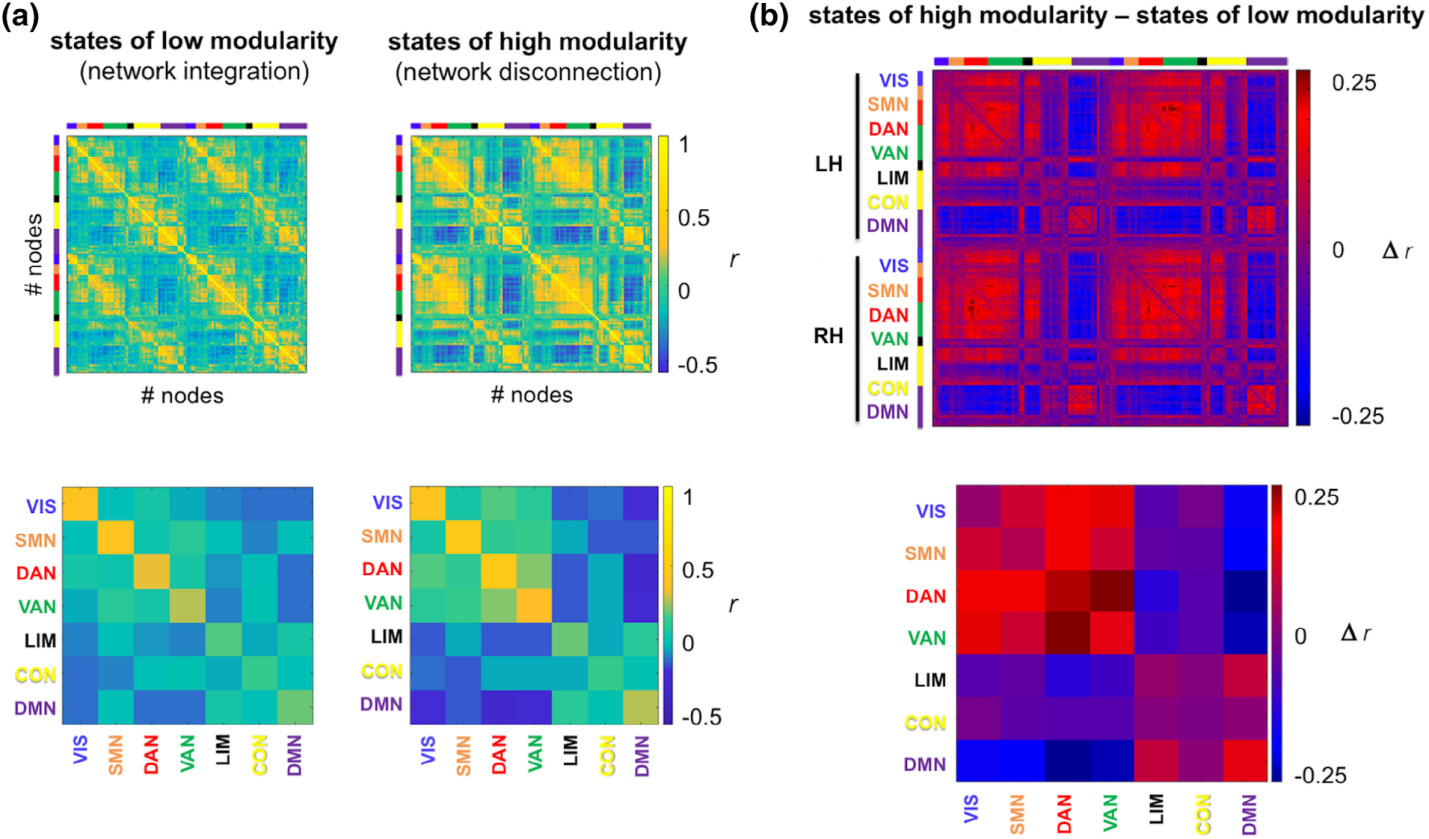
Extreme modularity states. (a) Group‐averaged functional connectivity profiles for states of particularly high or particularly low modularity. States were defined in relation to the across‐subject mean *Q*_*group*_ and are based on a fixed resolution level of *γ* = 35. For illustration, each node was assigned to a standard seven‐network partition (see Section 2), as depicted in different colors along the axes. (b) Difference in group‐averaged functional connectivity profiles between states of high and low modularity. For illustration of network‐specific connectivity values (bottom row), nodal connectivity values were aggregated within and between the seven standard networks (integrated across both hemispheres, see Section 2). CON, control network; DAN, dorsal attention network; DMN, default‐mode network; LH, left hemisphere; LIM, limbic network; *r*, Pearson's correlation coefficient; RH, right hemisphere; SMN, somatomotor network; VAN, ventral attention network; VIS, visual network [Color figure can be viewed at wileyonlinelibrary.com]

The increased stability of modular network organization in more intelligent persons may be related to the frequency at which the respective person's functional brain network resides in certain states of modularity, that is, states of particularly high or low modularity. In periods of low modularity, functional connectivity is more uniformly distributed across networks and is thought to reflect greater network integration (Shine, Bissett, et al., 2016). In contrast, periods of high modularity represent states of greater network segregation in the form of higher connectivity (more positive and coherent correlations) within modules and lower connectivity between different modules (more negative correlations, i.e., anticorrelations). This becomes also visible in our data as illustrated in the group‐averaged connectivity profiles for high‐ versus low‐modularity states (Figure 3a).

To explore whether the frequency of those states relates to general intelligence, we identified, per subject, states of particularly high or low modularity (i.e., time windows with *Q*_*ind*_ > mean *Q*_*ind*_ + 50% of mean *Q*_*ind*_ or *Q*_*ind*_ < mean *Q*_*ind*_ − 50% of mean *Q*_*ind*_) and tested across subjects whether the count of occurrences of these states correlated with intelligence. The results revealed significant negative associations between intelligence and the prevalence of high‐ and low‐modularity states (high *Q*_ind_ states: *rho* = −.14, *p* = .020, *R* ^*2*^ = .02; low *Q*_ind_ states: *rho* = −.12, *p* = .047, *R* ^*2*^ = .01; uncorrected for multiple comparisons; see also Figure 2b). As the figure shows, only a minority of subjects showed such subject‐specific states of high (*N* = 36) or low (*N* = 10) modularity. Post‐hoc analyses revealed that subjects with extreme modularity states were characterized by a relatively lower intelligence score: We observed significantly lower FSIQ values in subjects demonstrating high‐modularity states as compared to subjects demonstrating no such states (Mann–Whitney *U*‐test, two‐tailed: *z* = −2.236, *p* = .025; see Figure S2a) and a trend toward lower FSIQ values in subjects demonstrating low‐modularity states as compared to subjects demonstrating no such states (Mann–Whitney *U*‐test, two‐tailed: *z* = 1.657, *p* = .098; see Figure S2b). Given that functional connectivity estimates can be seriously affected by confounding effects of head motion (Ciric et al., 2017; Power et al., 2012, 2014; Siegel et al., 2017), we also examined whether subjects with extreme modularity states differed in respect to in‐scanner head motion (mean *FD*). However, no such effect was found (subjects demonstrating high‐modularity states versus subjects without such states, Mann–Whitney *U*‐test, two‐tailed: *z* = 0.214, *p* = .830; subjects showing low‐modularity states versus subjects without such states, Mann–Whitney *U*‐test, two‐tailed: *z* = −0.014, *p* = .989).

To obtain a measure for high‐/low‐modularity states that is more comparable between subjects, we repeated this analysis by defining states of high and low modularity relative to the group mean modularity *Q*_*group*_, which was defined by first averaging *Q*_*ind*_ across all participants and then *Q*_*group*_ across time windows (mean *Q*_*group*_ = 0.29). States of high modularity were defined, for each participant, as those time windows in which the individual modularity *Q*_*ind*_ exceeds the threshold of mean *Q*_*group*_ + 50% of mean *Q*_*group*_. This resulted in *Q*_*ind*_ > 0.36 as threshold for high‐modularity states. In contrast, states of low modularity were specified as below the threshold of mean *Q*_*group*_ − 50% of mean *Q*_*group*_. This resulted in *Q*_*ind*_ < 0.22 as threshold for low‐modularity states. We again observed a negative correlation between intelligence and the number of high‐modularity states (*rho* = −.12, *p* = .039, *R* ^*2*^ = .01; uncorrected), but no correlation for low‐modularity states (*rho* = −.04, *p* = .477; Figure 2c).

As we observed that the individual, time‐averaged dynamic modularity levels (i.e., modularity values computed for each time window separately and averaged afterward, different from modularity in static networks, see Section 3.1 static modularity) varied between individuals (time‐averaged *Q*_*ind*_: *M* = 0.24; *SD* = 0.05; range = 0.10–0.42) and as these variations may cause more or less frequent occurrences of extreme modularity states defined relative to the group mean modularity, we tested post‐hoc for potential associations between these individual, time‐averaged dynamic modularity levels and intelligence. There was no significant association (*rho* = .00, *p* = .956). Further post‐hoc analyses revealed that the reported effects were to some extent sensitive to the exact threshold chosen for the definition of high‐ versus low‐modularity states, which we infer from the observation that associations reach significance only within a certain range of *Q* thresholds (individual‐specific states: *Q*_*ind*_ *>* mean *Q*_*ind*_ + 5–60% of mean *Q*_*ind*_ for high‐*Q* states, *Q*_*ind*_ < mean *Q*_*ind*_ − 48–86% of mean *Q*_*ind*_ for low‐*Q* states; group‐averaged states: *Q*_*group*_ > mean *Q*_*group*_ + 50–56% of mean *Q*_*group*_ for high‐*Q* states, no sign. Effects for low‐*Q* states). Nevertheless, the direction of the relation remained unchanged across all percentiles and was the same for states defined relative to individual and group‐averaged mean modularity. In sum, both analyses suggest lower rates of high‐modularity states in subjects with higher intelligence scores.

### 3.3 Network‐specificity of intelligence‐stability association

Next, we computed the difference matrix (Figure 3b) between the group‐averaged connectivity profiles for high‐ and low‐modularity states (defined relative to the group mean modularity, *Q*_*group*_). By annotating this difference matrix with a canonical seven‐network partition (Yeo et al., 2011), we determined that the increased segregation during high‐modularity states is primarily driven by a stronger segregation (depicted in blue in Figure 3b) of brain regions that typically demonstrate decreased activation during tasks (DMN; Raichle et al., 2001) from brain regions associated with increased activation during task (VIS, DAN, and VAN).

**Figure 4:**
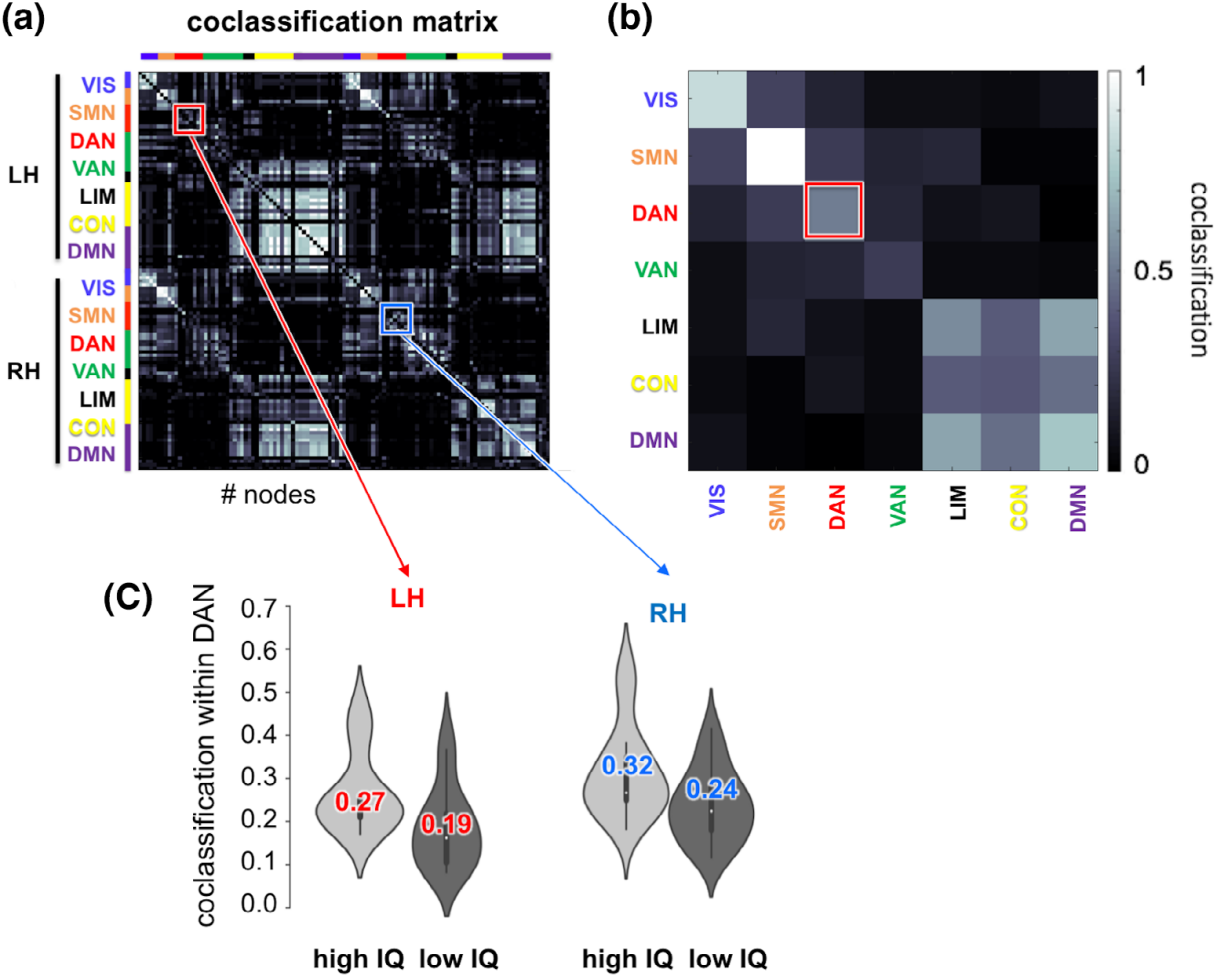
Results of coclassification analyses. (a) Group‐averaged coclassification matrix representing connection‐specific temporal stability scores (see Figure 1d). Values represent the proportion of time windows in which a given node pair is assigned to the same module. (b) Network‐specific stability values were calculated by averaging all connection‐specific coclassification scores within and between each of seven functional networks (aggregated across both hemispheres) provided by the Yeo atlas (Yeo et al., 2011). (c) Intelligence‐related effects in coclassification values of nodes within the dorsal attention network, depicted for those 28 subjects with the highest and lowest IQ (colored number, mean; white dot, median; thick bar, interquartile range). CON, control network; DAN, dorsal attention network; DMN, default‐mode network; IQ (FSIQ), Full Scale Intelligence Quotient assessed with Wechsler Abbreviated Scale of Intelligence (WASI; Wechsler, 1999); LH, left hemisphere; *r*, Pearson's correlation coefficient; LIM, limbic network; RH, right hemisphere; SMN, somatomotor network; VAN, ventral attention network; VIS, visual network [Color figure can be viewed at wileyonlinelibrary.com]

Finally, we aimed to investigate whether the association between higher intelligence and more stable network modularity over time is (a) driven by less variable segregation between all functional networks (higher consistency of all networks), (b) driven by less variable segregation between only some specific networks (higher consistency of specific networks), or (c) driven by less variable segregation between some specific networks that overrides rare periods of greater variability in segregation between other networks (higher consistency of specific networks that counts heavier than the lower consistency of other networks). To discriminate between these possibilities, we computed connection‐specific coclassification scores as the proportion of time windows in which a given node pair is assigned (coclassified) into the same module, with nodal network membership defined on the basis of the annotation with the canonical seven‐network partition (Yeo et al., 2011; Figures 3a,b and 4a,b). The resulting values are represented in the coclassification matrix (Figure 4a). Here, high values indicate that the two nodes of a given node pair are assigned to the same module most of the time (exhibiting stable consistent module affiliation), whereas low values indicate that a given node pair is only rarely coclassified as belonging to the same module (nodes stay consistently segregated from each other which may be interpreted as “stable absence” of segregation).

Network‐specific stability (Figure 4b) was determined by averaging the stability values (coclassification scores) of all connections within and between the seven standard networks. A network‐specific coclassification score of 1 would reflect that a given network (e.g., the VAN) is highly coherent internally (and thus very segregated from nodes in other modules). In contrast, a network‐specific coclassification score of 0 would reflect that a given network is totally incoherent and does not exist as a separable module. We thus interpret relatively higher coclassification scores for any given network as an indication of higher internal coherence of that respective network, that is, more segregation of that network from nodes in other networks, while lower coclassification indicates its dissolution.

In participants with higher intelligence scores, only nodes within the DAN were more consistently co‐assigned to the same module (positive correlation between intelligence scores and network‐specific coclassification scores:*rho* = .19, *p* = .0017, *R* ^*2*^ = .04; Bonferroni‐corrected threshold for 28 comparisons: *p* = .0018; Figure 4c). This result indicates that the DAN is internally more coherent (representing a more separable module) in people with higher intelligence scores than in people with lower intelligence scores. No other functional network demonstrated a significant association with intelligence (−.11 < *rho* ≤ .13; all *p* > .03). In sum, these results suggest that the global association between intelligence and more stable network modularity over time (see Section 3.2) is driven by a more coherent (and in that sense more stable) organization within a specific network, that is, the DAN. This conclusion is consistent with the above‐specified case (b).

## 4 DISCUSSION

We have shown that human intelligence is associated with the dynamic reconfiguration in functional brain networks as indexed by temporal fluctuations in global modularity. Participants with higher intelligence scores demonstrated higher stability of network segregation over time and exhibited lower rates of high‐modularity states. Our results suggest that an intrinsic network architecture exhibiting fluctuations within a more narrow range of modularity may offer an advantage in the face of momentary task‐driven demands related to cognition. Finally, we found that greater network stability associated with higher intelligence was driven primarily by brain regions belonging to the DAN.

### 4.1 Higher network stability associated with intelligence

Although previous research indicates that integrated and segregated information processing are both essential for human cognition (Cohen & D'Esposito, 2016), neither the general level of network integration (indexed by global efficiency; Hilger et al., 2017a; Kruschwitz et al., 2018) nor the general level of network segregation (indexed by global modularity; Hilger et al., 2017b, and present results) seem to differentiate between high versus low general intelligence — when investigated in static, time‐invariant networks. Rather, we observed here that higher intelligence is associated with more stable (i.e., less variable) levels of network segregation over time. Furthermore, we found that across the ~10 min of the task‐free fMRI scan, individuals with higher intelligence scores settled relatively less often into states of particularly high network segregation. A similar association for low segregation states was, however, only observed when defining these states relative to subject‐specific modularity thresholds. This latter result was driven by only a small subgroup of subjects and should therefore be interpreted with caution, requiring further investigation.

Studies of task fMRI suggest that states of low modularity facilitate network integration (Shine, Bissett, et al., 2016), probably because information can be exchanged more freely across module boundaries (Betzel et al., 2016), which is especially important for complex cognitive tasks requiring the coordination of different subprocesses (Cohen & D'Esposito, 2016; Shine, Bissett, et al., 2016). In contrast, high modularity facilitates network segregation. This is more characteristic of specialized information processing (Betzel et al., 2016), for example, in tasks requiring the unhindered processing of one type of information (e.g., motor information during finger tapping; Cohen & D'Esposito, 2016).

Additional support for the behavioral relevance of network segregation as indexed by modularity comes from clinical studies. Increased modularity has, for example, been observed in persons with Attention Deficit Hyperactivity Disorder (ADHD; Lin et al., 2014) or in patients suffering from major depression (MD; Ye et al., 2015). Graph‐theoretical investigations indicate that enhanced levels of network segregation can lead to a fragmented network organization with sharply isolated modules (Watts & Strogatz, 1998), which may cause a breakdown of communication between major functional subsystems. Interestingly, however, these two clinical conditions present with opposing cognitive deficits: while ADHD is associated with high levels of impulsivity (White, 1999), the executive function deficits observed in MD are associated with reduced cognitive flexibility (Lee, Hermens, Porter, & Redoblado‐hodge, 2012). These studies, however, are difficult to compare to this study because of methodological differences, including (a) that they relied primarily on static functional networks, (b) involved group comparisons that, accordingly, do not require the definition of concrete thresholds for high versus low modularity, and (c) differences in graph analysis methods (e.g., binary vs. weighted graphs).

Irrespective of the specific task content, the brain seems to decrease its general level of network segregation when switching from rest to task (Shine, Bissett, et al., 2016)—with lower levels of network segregation associated with higher cognitive performance (Cohen & D'Esposito, 2016; Shine, Bissett, et al., 2016). Based on recent evidence demonstrating that, during rest, intelligence is not per se associated with the level of segregation or integration (Hilger et al., 2017a, 2017b; Kruschwitz et al., 2018; Pamplona et al., 2015), one can plausibly assume that more intelligent people may invest more effort into reconfiguring their network when switching from rest to task in order to reach better‐suitable network configurations that facilitate high cognitive performance (Cohen & D'Esposito, 2016; Shine, Bissett, et al., 2016). The results of a recent study, however, point into exactly the opposite direction. Here, fewer differences between resting‐state and task‐general network organization were associated with higher levels of general intelligence—which the authors interpreted as indicating that more intelligent subjects need to reconfigure their network less when switching from rest to task (Schultz & Cole, 2016). This study adds a missing piece into this emerging picture as it reveals that during rest (a) higher temporal stability of intrinsic network segregation and (b) fewer states of extremely high network segregation are associated with higher levels of general intelligence.

Task‐related connectivity is assumed to rely critically on connectivity properties measured during rest (see also Amico, Arenas, & Goñi, 2019; Tavor et al., 2016), reflecting individual differences (Cole, Bassett, Power, Braver, & Petersen, 2014). Furthermore, first evidence suggests that individual profiles of connectivity dynamics generalize between rest and task and may therefore represent a task‐invariant common characteristic (Fong et al., 2019). These observations suggest that the association between intelligence and higher stability in network organization over time is not limited to task‐free (resting‐state) conditions, but represents a more general phenomenon. Against this background, we here speculate that during cognitive tasks (with unchanging cognitive demands), higher intelligence may be associated with both, that is, a more effectively reduced degree of network segregation (Cohen & D'Esposito, 2016; Shine, Bissett, et al., 2016), and an overall higher extent of temporal stability within this adapted architecture (this study and Fong et al., 2019). Finally, the association between intelligence and fewer occurrences of high‐modularity states may suggest an intrinsic protection against unintentional shifts toward states of network fragmentation (i.e., particularly high modularity) that are likely to disrupt information processing and hinder ongoing cognition.

Our conclusion is complementary to a recent proposal suggesting that general intelligence depends on the ability to flexibly transition between “easy‐to‐reach” and “difficult‐to‐reach” network states (Barbey, 2018; Girn, Mills, & Christo, 2019). Our finding of higher intelligence associated with greater temporal stability in network organization during rest (see also Fong et al., 2019, for similar finding under task conditions) expands on the intuitively plausible proposal that higher intelligence relies on higher task‐dependent network flexibility. We thus propose that higher intelligence may be associated with both, that is, higher flexibility in network configurations when task demands change, and higher network stability when task demands remain stable across time. The latter may occur when subjects engage in the same task (Fong et al., 2019) or remain within the resting‐state condition (this study and in Fong et al., 2019). However, this suggestion needs to be tested empirically by studies investigating dynamic changes in network organization that occur during the switch from rest into task. The proposal of Barbey (2018) postulates further that the superior “ability” of more intelligent people to adaptively form task‐specific network configurations results from differences in intrinsic small‐world network attributes, specifically, in global network integration or global network segregation levels measured in static functional brain networks (Girn et al., 2019). Contrasting this view, we observed no relation between global modularity and intelligence in static, time‐averaged network metrics (see also Hilger et al., 2017b).

### 4.2 Dorsal attention network as locus of intelligence‐related network stability

Finally, we identified the DAN as primary locus of the observed association between intelligence and temporal brain network stability. Previous research has associated the DAN with controlled and voluntarily reorientation of attention toward goal‐relevant information (Corbetta & Shulman, 2002) — a process that is involved in many cognitive tasks and has been linked to general intelligence (Engle, 2018; Schweizer, Moosbrugger, & Goldhammer, 2005). Also functional‐neuroanatomical considerations suggest a specific role for the DAN in intelligence, given that both functional and structural correlates of intelligence have been revealed in this system (Basten et al., 2015; Jung & Haier, 2007). In a meta‐analysis of fMRI studies (Basten et al., 2015), we found across‐study overlap of intelligence‐related activation effects in the superior parietal lobe, precuneus, frontal regions including the frontal eye fields and precentral ventral frontal cortex, and in the middle temporal gyri. These brain regions partly overlap with the DAN (Yeo et al., 2011). In addition, two recent studies also support the idea that the way in which attention‐related brain regions are embedded into the intrinsic functional network topology differentiates between persons with higher and lower intelligence scores (Hilger et al., 2017a, 2017b). However, these two studies investigated intelligence‐related effects in static connectivity and associations were observed in regions primarily associated with bottom‐up (stimulus‐driven) attention (ventral attention network). This inconsistency indicates that different insights may be gained from static as opposed to dynamic network features (Zalesky et al., 2014) and stresses the importance of considering both dimensions when trying to understand comprehensively how different network architectures may contribute to individual differences in cognition. This gap was addressed in a very recent study revealing that higher stability in functional brain network organization during rest and task was associated with better performance in a variety of tasks requiring controlled attention (Fong et al., 2019).

### 4.3 Limitations

Our study was designed to test whether intelligence is associated with brain network dynamics and our results reveal a significant association, specifically, a more stable network organization in people with higher intelligence scores. However, it must be acknowledged that the amount of variance explained by these dynamic network properties seems to be small (i.e., around 4%), especially in comparison with recent studies using predictive machine learning approaches. Finn et al. (2015) report that up to 25% of variance in intelligence can be explained by a multivariate combination of 71,824 intrinsic connectivity values spanning across the whole brain. Subsequent evidence, however, has been heterogeneous with some studies reporting prediction results of only around 5% explained variance despite using a similar predictive statistical approach (Ferguson, Anderson, & Spreng, 2017). In the current study, we followed a different approach: Rather than using all possible combinations of connectivity values, we instead tested for a potential relation between intelligence and only one metric (i.e., *SD* of *Q* over time), which has been suggested to capture fundamental aspects of dynamic network organization. Therefore, we did not expect as high amounts of explained variance as observed in multivariate predictive approaches. Nevertheless, we would like to stress that current findings of associations between brain network topology and intelligence should be interpreted with caution, as a more comprehensive and definitive understanding of this relationship will require additional study, including work combining static and dynamic functional connectivity features.

Another important issue is head motion, which has been shown to significantly influence estimates of functional connectivity in static networks (Ciric et al., 2017; Power et al., 2014) and can bias associations with behavioral measures (Siegel et al., 2017). Although it has been suggested that dynamic (time‐varying) functional connectivity is relatively insensitive to head motion (see, e.g., Abrol et al., 2017, who report high replicability for dynamic connectivity), and although we controlled for head motion (mean *FD*) in all analyses, we cannot ultimately rule out the existence of remaining unknown influence of head motion on our connectivity estimates.

## 5 CONCLUSION

Taken together, our results reveal that the temporal stability of modular brain network organization is associated with individual differences in a person's general capacity for cognition and intellectual performance. Cognition requires both integrated and segregated information processing, and the human brain has been shown to flexibly adapt its functional network architecture to meet different task demands. The results of our study suggest that when taking into account the temporal dynamics of network organization, a more stable level of network segregation over time is associated with higher levels of cognitive performance. We conclude that such an organization may constitute an optimal foundation for focused task processing and may protect the brain against the occurrence of maladaptive network states. The locus of our results within the DAN suggests specific relevance of brain regions associated with controlled top‐down attention to maintain this intelligence‐related advantage of higher network stability. Taken together, our study proposes that the investigation of brain network dynamics may have great potential to refine our understanding about the mechanisms underlying human intelligence.

## CONFLICT OF INTEREST

The authors declared no conflict of interest.

## AUTHOR CONTRIBUTIONS

K.H., C.F., and O.S. conceptualized the study. K.H. managed the data. M.F. preprocessed the data and provided theoretical help. K.H. and O.S. analyzed the data. K.H., C.F., and O.S. interpreted the results and wrote the manuscript.

## Supporting information


**Figures S1 and S2**: Supporting information

## Data Availability

All data used in the current study can be accessed online under: http://fcon_1000.projects.nitrc.org/indi/enhanced/. The preprocessing pipeline CCS is also freely available to the public via GitHub (https://github.com/zuoxinian/CCS) or http://lfcd.psych.ac.cn/ccs.html. All further analysis code used in the current study has been deposited on GitHub (https://github.com/KirstenHilger/Dynamic‐Brain‐Network‐Modularity) and Zenodo (https://zenodo.org/record/2918712).
