# Supplementary material for: Temporal stability of functional brain modules associated with human intelligence
Source: Hum Brain Mapp. 2019 Oct 6;41(2):362–72. doi: 10.1002/hbm.24807 (PMC7267930; doi:10.1002/hbm.24807)
Supplement: Supplementary file 1 — Figures S1 and S2: Supporting information [file HBM-41-362-s001.pdf]

SUPPORTING INFORMATION

Temporal Stability of Functional Brain Modules Associated with Human Intelligence

Kirsten Hilger[1,2,*], Makoto Fukushima[3], Olaf Sporns[3,a], Christian J. Fiebach[1,2,4,a]

[1] Department of Psychology, Goethe University, Theodor-W.-Adorno-Platz 6, 60323 Frankfurt am Main, Germany

[2] IDeA Center for Individual Development and Adaptive Education, Rostocker Str. 5, 60323 Frankfurt am Main, Germany

[3] Department of Psychological and Brain Sciences, Indiana University, Psychology Building 360, 1101 E 10th Street, Bloomington, IN 47405, USA

[4] Brain Imaging Center, Goethe University Frankfurt, Schleusenweg 2-16, 60528 Frankfurt am Main, German

[a] These authors should be considered joint senior author

ORCID:
Kirsten Hilger: 0000-0003-3940-5884
Makoto Fukushima: 0000-0003-2345-2598
Olaf Sporns: 0000-0001-7265-4036
Christian J. Fiebach: 0000-0003-0827-1721

[*] <u>Corresponding author:</u>
Kirsten Hilger
Goethe University Frankfurt, Department of Psychology
Theodor-W.-Adorno-Platz 6, PEG
D-60323 Frankfurt am Main
Phone: +49 (0)69 / 798 - 35345
hilger@psych.uni-frankfurt.de

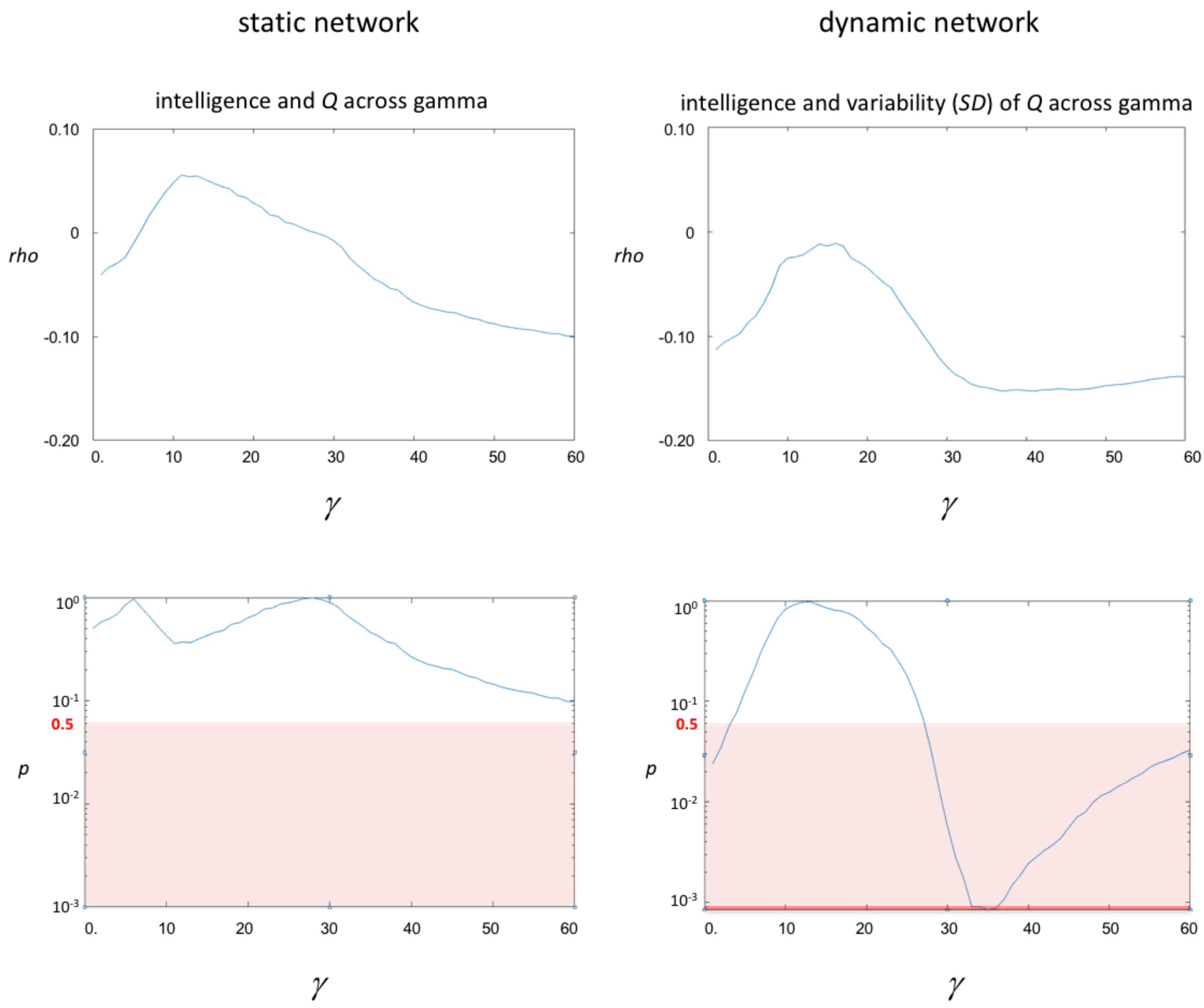

FIGURE S1 Associations between intelligence and (variability of) global modularity across varying resolution levels. *Left:* Association between intelligence and global modularity *Q* in static (i.e., time-averaged) networks, for different levels of the network resolution parameter gamma (γ; *x*-axis). *Right:* Association between intelligence and the variability in global modularity (operationalized as standard deviation of Q_{ind} across time) in dynamic networks, displayed across gamma (γ) levels. *Bottom row:* Significance levels (*p*-values, uncorrected for multiple comparisons) across different network resolutions (gamma levels); area indicating *p*-values < .05 is marked in red; area indicating *p*-values < .00083 (Bonferroni corrected for 60 multiple comparisons) is marked in dark red. *Q*, global modularity of individual-specific module partitions; *SD*, standard deviation; *rho*, Spearman correlation coefficient for the partial-correlation between intelligence and (*SD* of) *Q* after controlling for effects of age, sex, handedness, and mean framewise displacement; γ, resolution parameter gamma (adjusting size/number of modules received from modularity maximization).

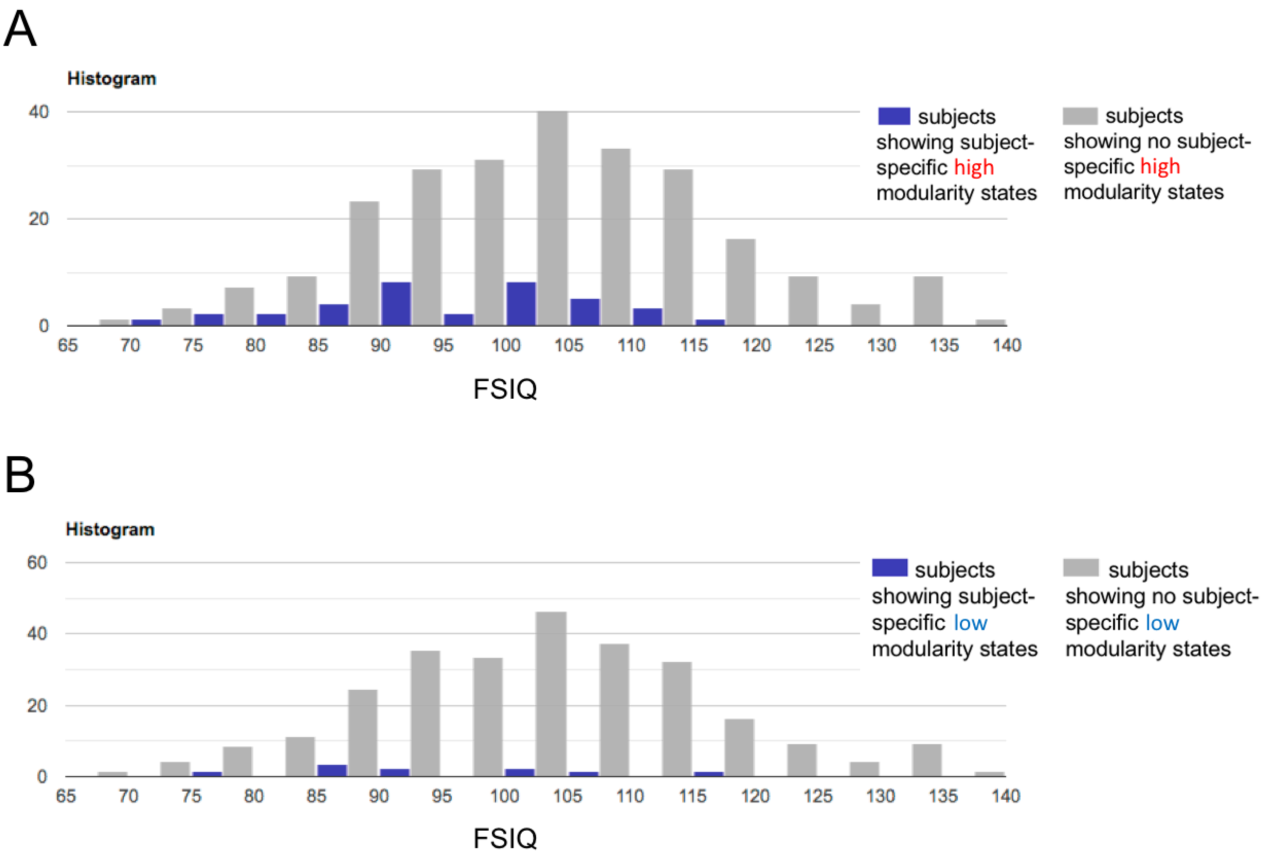

FIGURE S2 Histograms of intelligence scores for subjects demonstrating subject-specific states of (*A*) extreme high modularity and (*B*) states of extreme low modularity (blue) in comparison with subjects demonstrating no such states (grey).
